# Rapid chemokinetic movement and the invasive potential of lung cancer cells; a functional molecular study

**DOI:** 10.1186/1471-2407-6-151

**Published:** 2006-06-07

**Authors:** Sandra YY Fok, Jeffrey S Rubin, Fiona Pixley, John Condeelis, Filip Braet, Lilian L Soon

**Affiliations:** 1 Electron Microscope Unit, Australian Key Centre for Microscopy and Microanalysis, University of Sydney, Sydney, NSW, 2006, Australia; 2 Laboratory of Cellular and Molecular Biology, NCI, National Institutes of Health, Bethesda, MD 20892, USA; 3 Department of Anatomy and Structural Biology, Albert Einstein College of Medicine of Yeshiva University, Bronx, NY 10461, USA

## Abstract

**Background:**

Non-small cell lung cancer is the most common cause of early casualty from malignant disease in western countries. The heterogeneous nature of these cells has been identified by histochemical and microarray biomarker analyses. Unfortunately, the morphological, molecular and biological variation within cell lines used as models for invasion and metastasis are not well understood. In this study, we test the hypothesis that heterogeneous cancer cells exhibit variable motility responses such as chemokinesis and chemotaxis that can be characterized molecularly.

**Methods:**

A subpopulation of H460 lung cancer cells called KINE that migrated under chemokinetic (no gradient) conditions was harvested from Boyden chambers and cultured. Time-lapsed microscopy, immunofluorescence microscopy and microarray analyses were then carried out comparing chemokinetic KINE cells with the unselected CON cell population.

**Results:**

Time-lapsed microscopy and analysis showed that KINE cells moved faster but less directionally than the unselected control population (CON), confirming their chemokinetic character. Of note was that chemokinetic KINE cells also chemotaxed efficiently. KINE cells were less adhesive to substrate than CON cells and demonstrated loss of mature focal adhesions at the leading edge and the presence of non-focalized cortical actin. These characteristics are common in highly motile amoeboid cells that may favour faster motility speeds. KINE cells were also significantly more invasive compared to CON. Gene array studies and real-time PCR showed the downregulation of a gene called, ROM, in highly chemokinetic KINE compared to mainly chemotactic CON cells. ROM was also reduced in expression in a panel of lung cancer cell lines compared to normal lung cells.

**Conclusion:**

This study shows that cancer cells that are efficient in both chemokinesis and chemotaxis demonstrate high invasion levels. These cells possess different morphological, cytoskeletal and adhesive properties from another population that are only efficient at chemotaxis, indicating a loss in polarity. Understanding the regulation of polarity in the context of cell motility is important in order to improve control and inhibition of invasion and metastasis.

## Background

Non-small cell lung cancer (NSCLC) is the most common cause of early casualty from malignant disease in western countries and is classified into three main histological subtypes: adenocarcinomas (AC), squamous cell carcinomas (SCC), and large cell carcinomas [1]. The heterogeneous nature of NSCLC has been documented by both histochemical and microarray analyses. Gene expression profiling indicated that ACs are the most heterogeneous type and can be further separated into three [2] or four subgroups [3]. Ultrastructural and immunocytochemical analyses showed that 92% of NSCLC had undergone at least one differentiation event while 27% demonstrated double differentiation events: adenosquamous and adeno-neuroendocrine [4]. Lung cancers are therefore, subject to differentiation and are morphologically and molecularly heterogeneous. In all likelihood, lung cancer cell lines derived from tumor specimens are also heterogeneous in nature. There is, however, little known about the morphological, molecular and biological variation within cell lines used as models for invasion and metastasis.

Cancer cells disseminate from primary tumor sites utilizing different modes of motility and invasion, including protease-independent amoeboid crawling, and integrin- and protease-dependent mesenchymal migration [5-7]. Cell motility is distinguishable by response to external factors. Chemotaxis in the context of cancer is directed cell motility towards diffusible factors. Chemokinesis however, is motility in response to soluble factors in the absence of a gradient, involving a change in speed or turning behaviour. Furthermore, cell motility is generally studied in two ways; using end-point Boyden chambers [8] and time-lapse microscopy that allows recording and analysis of cell motility [9].

The response of cells to growth factors incorporates both chemokinesis and chemotaxis [10]. Chemokinesis may play a role during the process of epithelial-mesenchymal transition (EMT), facilitating the separation of tumor cells from the tumor mass through autocrine signals [11]. Chemotaxis, on the other hand, has an important role in homing mechanisms [12]. In these studies, however, it was unclear whether the observations were representative of a homogenous population or whether they reflected a heterogeneous population where, for instance, some cells were more chemotactic and others more chemokinetic. We hypothesize that some cancer cell lines are heterogeneous exhibiting different types motility based on the premises that (1) the type of motility depends on the cellular architecture regulated by the cytoskeleton and, (2) some cells lines demonstrate variability in cell shape and polarity, indicating differences in cytoskeletal organization, matrix adhesion, and likely cell motility.

In this study, we isolated and characterized a population of lung cancer cells designated KINE cells that showed enhanced chemokinesis compared to unselected CON cells. The two populations were evaluated for motility using Boyden chambers and time-lapse microscopy, which confirmed that KINE cells were chemokinetic. They were also less adhesive to substrate and were more invasive compared to CON cells. Evaluation of gene expression by microarray studies indicated differential gene expression patterns between the two populations with implications in chemokinesis and invasiveness.

## Methods

### Cell culture

H460 lung cancer cells (ATCC) were cultured in Dulbecco's modified Eagle's medium (DMEM) (Invitrogen Corp., Carlsbad, California) supplemented with 10% fetal bovine serum (FBS) (Invitrogen), penicillin and streptomycin (Invitrogen).

### Migration and invasion assays

Migration assays were carried out as previously described [8]. In brief, 2 × 10^4 ^cells from overnight cultures were placed in upper migration chambers (Beckton Dickinson, Bedford, MA). In chemokinetic assays, both upper and lower chambers were filled with 1.5% serum in DMEM whereas for chemotactic assays, only the lower chamber contained 1.5% serum. Various combinations of serum in upper and lower chambers were used for checkerboard analyses (Table 1). Incubation was carried out for 8 h in a humidified CO_2 _chamber and cells on the lower surface of the filter were then stained with methylene blue. The number of migrated cells were counted from a total of nine regions on the filter and calculated as numbers/cm^2^. Invasion assays were carried out in chambers coated with a synthetic matrix layer, ECMatrix (Chemicon). A total of 1 × 10^5 ^cells in 1.5% FBS were placed in the upper chambers and the lower chamber was filled with 10% FBS in DMEM. The assay was carried out for 48 h and then processed as described above.

### Isolation of chemokinetic cells

**Figure 1:**
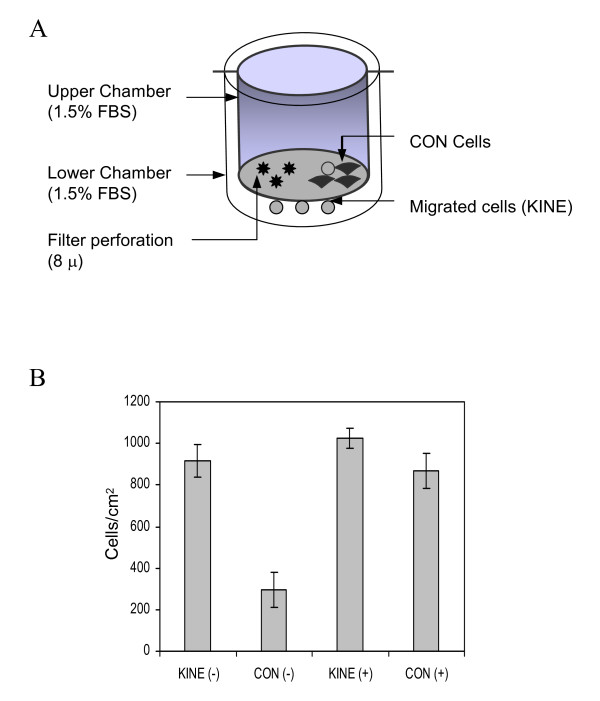
**Selection and enrichment for chemokinetic H460 cancer cells**. **(A) **Boyden chamber assays were used with 1.5 % serum present in both upper and lower chambers. Cells that migrated across the filter was collected by trypsinization and cultured to expand their numbers. These cells were further enriched for chemokinetic KINE cells in similar experiments for a total of seven times with intermittent periods of 48 h to allow growth in full serum conditions. **(B) **Migration assays were conducted under chemotactic conditions in the presence of 1.5% fetal bovine serum in the lower chamber only (positive gradient), and under chemokinetic conditions in the presence of 1.5% serum in both upper and lower chambers (uniform gradient). The KINE population of cells demonstrated greater chemokinetic activity in the absence of a serum gradient compared to CON cells (CON). There was no difference in chemotaxis levels between the two populations (-) = Uniform serum gradient; (+) = Positive serum gradient. Data represent the mean ± S.D. of triplicate determinations.

A chemokinetic assay as described above was used to isolate chemokinetic cells. Instead of staining with methylene blue, the cells on the bottom of the filter were trypsinized and allowed to expand in numbers under viable (full serum), growth conditions for two days (Figure 1A). This process of chemokinesis, cell collection and expansion was repeated a total of seven times resulting in a population of cells that were highly chemokinetic.

### Digital microscopy and computer-assisted analysis of cell behavior

Cells were seeded on glass-bottom dishes (MatTek Corp., Ashland, MA) at a density of 0.5 × 10^4 ^cells per dish for 16 h [13]. The motility of cells was recorded for the duration of 1 h by time-lapse microscopy using a ×20 objective on an inverted microscope, a charge-coupled device camera (Cooke Corp., MI, USA), and IP Lab Spectrum software. Each sequence consisting of 60 frames, taken at 1 min intervals, was imported into IMAGEJ for reconstruction into movies. The cells were traced and each traced sequence was analyzed using Dynamic Image Analysis Software (DIAS) (Soll Technologies, Inc., Iowa City, IA USA), to obtain a series of motility parameters.

### Fluorescence microscopy

Immunofluorescence microscopy was carried out as previously described [14]. Cells were fixed for 5 min in 3.7% paraformaldehyde (Fisher Scientific, Springfield, NJ) and permeabilized with 0.5% Triton X-100 for 20 min. Following washes with Tris-buffered saline, pH 8.0 (TBS), the samples were treated with a solution containing Alexa-488-phalloidin conjugates (Molecular Probes, Eugene, OR), 1% BSA and 1% FBS in TBS. Anti-tyrosine-188 paxillin antibody was used to bind paxillin and a goat anti-mouse-Alexa-568 conjugate (Molecular Probes) was used as the secondary antibody for the localization and detection of the primary label. Image acquisition was conducted using a 60× objective on an Olympus microscope with filter sets in the excitation-emission range of the fluorophores (Olympus America Inc., Melville, NY).

### Cell adhesion assay

Cells were trypsinized, washed with Dulbecco's phosphate buffered saline (DPBS) and seeded at a density of 4 × 10^4 ^cells/ml in 500 μl of complete medium in 24-well plates for 24 h. A stock trypsin-EDTA solution (.05%) (Invitrogen Corp.) was use undiluted (100%) or diluted to 66%, 50% and 40% of the stock concentration. The cells were washed once in the diluted trypsin and incubated in fresh trypsin for 10 min at 37°C. All detached cells were collected following one wash with DPBS. The remaining cells were lifted by trypsinization and counted. The percentage of adherent cells was then calculated and graphed.

### Microarray analysis and real-time PCR

Microarray analyses were performed as previously described [8]. In brief, CON and KINE H460 cells seeded at equal density for 24 h were processed for RNA precipitation using RNeasy columns (Qiagen, Valencia, CA). The RNA was converted into double-stranded cDNA and finally biotinylated cRNA in a series of reactions (Affymetrix, Santa Clara, CA). The cRNA was fragmented and used in a hybridization reaction on U133A Affymetrix chips that were subsequently processed at a fluidics station. The probe array was scanned and the data analyzed using the Microarray Suite Software (Affymetrix). A rank-based statistical test and computation of the log ratio (2) of probe signal intensities identified genes that were differentially expressed between the two populations of cells.

The microarray data were verified by real-time PCR using a set of RNA preparations different from that used in the array. In real-time PCR studies, first-strand cDNA was generated by oligo-dT priming of RNA from KINE and CON cell populations (Roche). cDNA template derived from various lung cancer cell lines was also used in a reaction to determine relative levels of expression of ROM. The template and gene-specific primers were added to a PCR mix containing the SYBR green reporter molecule (Applied Biosystems, Foster City, CA) and the PCR reaction was carried out in the ABI Prism 7000 Sequence Detector (Applied Biosystems). The comparative cycle threshold (C_T_) method was used to analyze the data by generating relative values of the amount of target cDNA. C_T _values indicate the number of cycles during amplification of target to reach a fixed threshold and correlate with the amount of starting material present. To obtain relative values, the following arithmetic formula was used: 2^-ΔΔCT^, where ΔCT = difference between the threshold cycles of the target and an endogenous reference (actin), and -ΔΔCT = difference between ΔCT of the target sample and a designated calibrator (vector control). The calculated result represents the amount of normalized target relative to the calibrator.

### Microarray data

The data discussed in this publication have been deposited in NCBIs Gene Expression Omnibus [15] and are accessible through GEO accession numbers GSE4869, GSM109527 and GSM109528.

## Results

### Chemokinesis and chemotaxis of H460 lung cancer cells

The motility characteristics of the non-small cell lung cancer (NSCLC) cell line, H460, were investigated by checkerboard analysis using Boyden chambers. A spectrum of serum concentrations in both upper and lower chambers was used to generate positive or negative gradients across the filter as shown in Table 1. Analysis of the behavior of H460 cells indicated that they were highly chemotactic and migrated well in the presence of a serum gradient (Table 2); high levels of migration across the filter in the presence of serum within the lower chamber (Conditions B-D) illustrated efficient chemotaxis of these cells. Low levels of chemokinesis by a subpopulation of cells were also detected where cells migrated in the absence of a serum gradient (Conditions E, F, I-K and M-P) (Table 2).

### Enrichment for chemokinetic H460 cells

An iterative process of selection and culturing was used to isolate a subpopulation of H460 cells demonstrating chemokinesis. We recovered cells that exhibited chemokinesis under conditions where 1.5% serum was present in both upper and lower chambers. Chemokinetic conditions in the presence of serum were chosen over non-serum conditions to avoid stress induced by serum-starvation. A concentration of 1.5% was chosen because this serum level was sufficient to maintain cell survival and was shown to be effective for the assay. Chemokinetic cells were removed from the underside of the filter by trypsinization and were allowed to expand in numbers by culturing for two days in full serum (Figure 1A). This procedure (chemokinetic assay, cell collection and cell expansion) was repeated seven times to enrich for chemokinetic cells called KINE. The control population (CON) consisted of the original population of unselected cells. KINE and CON cells were then reanalysed in Boyden chamber experiments. The chemotactic environment was created by placing 1.5 % FBS in the lower chamber (+ve gradient). A chemokinetic environment was generated using 1.5 % FBS in both upper and lower chambers (-ve gradient). Figure 1B showed that KINE cells actively migrated across the filter irrespective of whether a 1.5 % serum gradient was present (+) or absent (-). CON cells, on the other hand, were more migratory in the presence of a serum gradient. KINE cells, therefore, were significantly more chemokinetic compared to CON cells but both populations of cells were efficient in their ability to chemotax (Figure 1B).

### Cell morphology and actin organization

**Figure 2:**
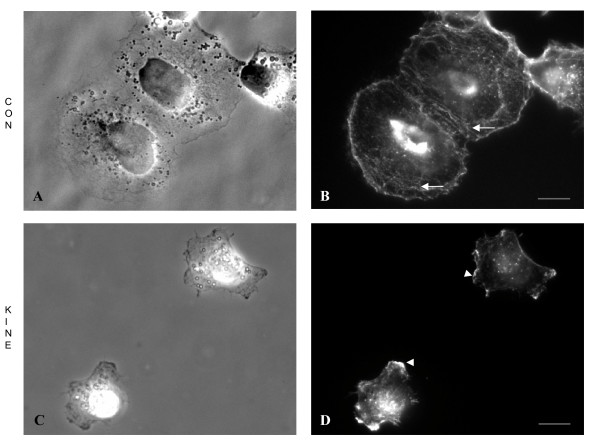
**Morphological analysis and actin organization of KINE and CON cells**. Cells were grown in complete medium for 24 h before fixation and processing for phase contrast and epifluorescence microscopy. **(A,B) **CON cells form rosettes or clusters and appeared more spread and polarized compared to **(C,D) **KINE cells, which rarely cluster. Alexa 488-Phalloidin staining of actin shows more prominent stress fibres in **(B) **CON (arrows) compared **(D) **KINE cells. KINE cells, however, possess actin-rich membrane ruffles at the leading edge (arrowheads) absent in CON cells. (Bars = 5 microns).

**Figure 3:**
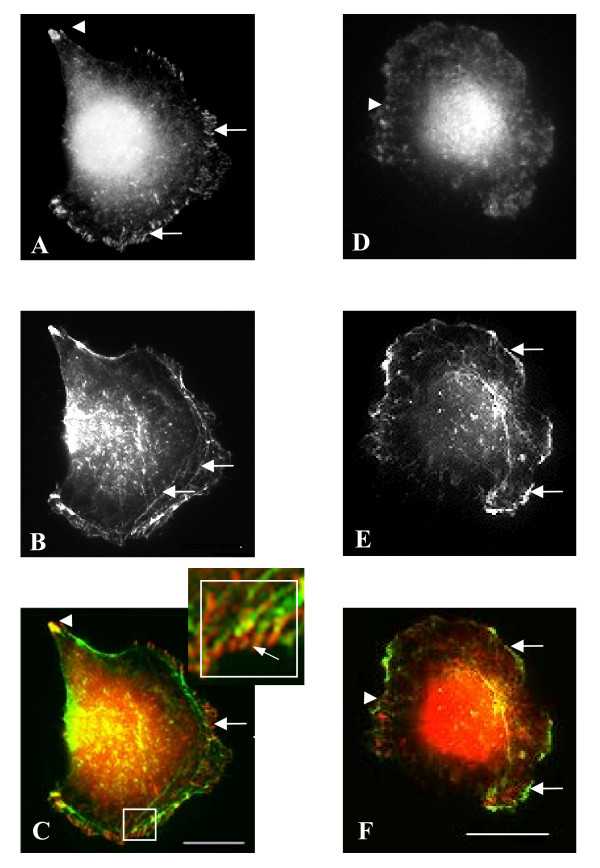
**Adhesion structures of CON and KINE cells**. Panels **A & D **show paxillin-y118 localization. Panels **B & E **show actin staining and Panels **C & F **show merged channels for paxillin-y118 and actin. Cells grown in complete medium were fixed and processed for the immunolocalization of paxillin-y118. CON cells stained for paxillin-y118 at focal adhesions at the tip of the uropod (arrowhead) and the leading edge (arrows) **(A, C)**. The focal adhesions at the leading edge were connected to internal actin cables **(B, C **and **inset)**. In KINE cells, however, focal adhesions were absent from the leading edge but less distinct focal complexes are present within the lamellae (arrowheads) **(D, F)**. The leading edge of KINE cells show many actin-rich ruffles (arrows) **(E, F) **(Bars = 5 microns).

The morphology and structural organization of KINE and CON cells were investigated by phase contrast microscopy and fluorescence staining for actin. Cells were fixed, stained with phalloidin-Alexa-488 conjugates, and processed for epifluorescence microscopy. CON cells appeared more spread and possessed polarized shapes whether as tight epitheloid-sheets (Figure 2A) or singularly whereby a leading edge and retracting uropod were present (Figure 3A). KINE cells, on the other hand, were less adherent to neighboring cells, appeared unpolarized without distinctive uropods, and possessed actin-rich membrane ruffles at the leading edge (Figures 2D, 3E).

### Cell adhesion, focal complexes and focal adhesions

Cell attachment to substrate is characterized by the formation of focal adhesions consisting of clusters of membrane-spanning integrins and intracellular molecules such as paxillin, FAK, talin and vinculin. The matrix adhesion structures of the lung cancer cells were investigated by immunofluorescence localization of a phosphospecific antibody to tyrosine-118 of paxillin. In CON cells, y118-paxillin localized to distinct focal adhesions at the retracting uropod (arrowhead) and at the leading edges of lamellae (arrows) (Figure 3A,C). Actin cables terminate at the leading edge where they superimpose with focal adhesions (Figure 3B,C and inset). In contrast, focal adhesions were absent at the leading edge of KINE cells. Instead, smaller, less distinct focal complexes were present in the lamellipodia of these cells (arrowhead) (Figure 3D,F). In addition, the leading edge of KINE cells was active with actin-rich ruffles (arrows) (Figure 3E,F).

### Adhesion to the substratum

**Figure 4:**
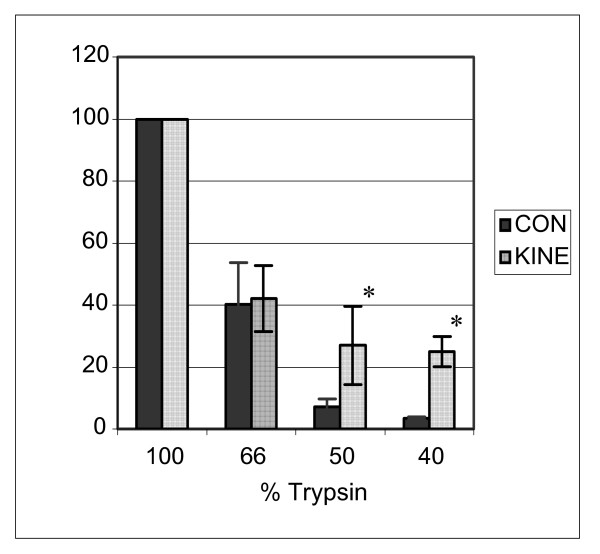
**KINE and CON cells were tested for strength of adhesion to substrate**. Adhesion assays were conducted by plating cells on uncoated tissue culture dishes followed by trypsinization for 10 min using various concentrations of tryspin. T-test at 5% confidence levels showed significant difference at lower concentrations of trypsin used (asterisks), indicating greater sensitivity to trypsinization in KINE compared to CON cells (n = 3). Conversely, the resistance of CON cells to trypsinization suggests stronger adhesion to substrate.

The rounded appearance of KINE cells and the lack of a distinct uropod (Figure 3D–F) is indicative of low polarity, cell tension and adhesiveness to the substratum. To investigate whether KINE cells differed from CON cells in their adhesive properties, an adhesion assay was conducted. This study investigated adhesion as an inherent property of cells that included modulation of their own adhesive environment through, for example, secretion of matrix proteins; for this reason the cell culture plates were left uncoated. Cells were grown for 24 h on 24-well plates and trypsinized for 10 min using a range of concentrations of trypsin. At low trypsin concentrations, CON cells were resistant to detachment from the culture plate whereas KINE cells detached in significantly greater numbers than CON cells (Figure 4).

### Digital image analysis of chemokinesis

The parameters of cell motility under chemokinetic/isotropic conditions in 2-dimensional cultures were analyzed for both KINE and CON cells. The cells were plated onto tissue culture dishes for 16 h and monitored under time-lapsed microscopy. Frames were obtained 1 min apart for 1 h and analyzed using the DIAS software. Student's t-test results reject the null hypothesis that there was no difference in the total path length, directionality, speed and area but not the net path length between KINE and CON cells. KINE cells achieved significantly greater total path length compared to CON cells, whereas the net path length was not significantly different between the cell lines (Table 3). The resulting calculation for directionality, a function of total path length divided by net path length, showed significant difference between KINE and CON cells. This indicated that CON cells moved more linearly than KINE cells, which made more random turns during the course of cell motility. The two cell types also showed a large difference in their migration speeds where KINE cells migrated approximately 30% faster than CON cells (Table 3). The greater total path length for KINE cells in isotropic medium reflected the results for Boyden chamber assays showing that they were more motile than CON cells in the absence of a gradient (Figure 1B).

### *In vitro *cell invasion

**Figure 5:**
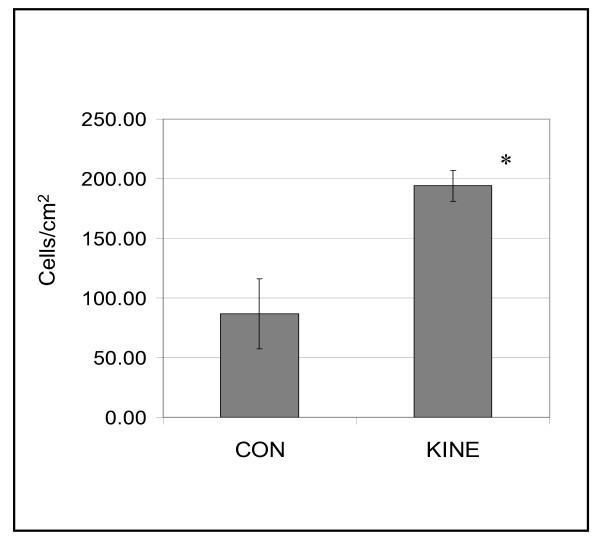
**In an *in vitro *invasion assay using reconstructed tumor basement membrane (Chemicon) as a substrate in chambered wells**. KINE cells were significantly more invasive compared to CON cells. (Data is representative of three experiments and indicate mean ± S.D. of triplicate samples. Significance value to <0.05).

To evaluate the correlation between increased chemokinesis and invasion, the invasive capacity of KINE and CON cells was examined in basement membrane-coated chamber wells. KINE and CON cells were trypsinized, washed in buffer, resuspended in 1.5% FBS containing DMEM, and placed in the upper chamber. The lower chamber was filled with 10% FBS in DMEM. The number of cells able to invade through the basement membrane was scored. KINE cells were significantly more invasive than CON, demonstrating a positive correlation between chemokinesis and cell invasion (Figure 5).

### Identification of ROM as a gene downregulated in chemokinetic cells

The two population of cells were analyzed in microarray hybridization studies to evaluate gene expression levels. Total RNA extracted from the cells was used for the generation of double-stranded cDNA followed by the production of biotinylated cRNA. The biotinylated probes were hybridized to U133A Affymetrix gene chips that were processed and scanned, and the data subjected to binary comparative analyses using the Microarray Suite Software. A rank-based statistical test and computation of the log ratio (2) of probe signal intensities identified genes that were differentially expressed between the two populations of cells.

**Figure 6:**
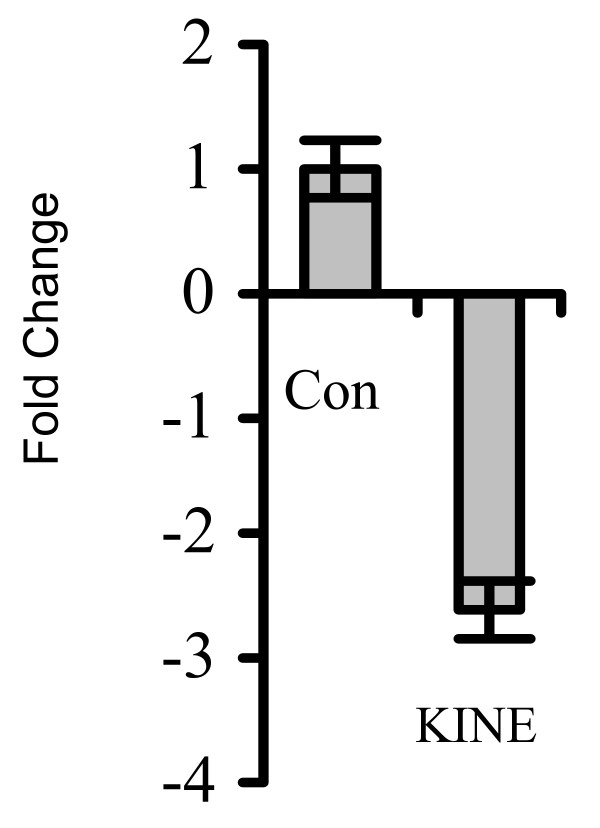
**Real-time PCR analysis of ROM expression in KINE and CON cells**. Real-time PCR was conducted to confirm data obtained from microarray analyses. Cycle threshold (C_T_) values were used to calculate the fold change of mRNA levels of ROM in KINE compared to CON cells. The data indicated that the expression of ROM was reduced in KINE cells by 3-fold over control.

A gene, known by the Affymetrix target, 209606, was lower in expression by approximately four-fold in KINE cells compared to the control. This gene, named reduced on-random motile (ROM) was further analyzed in subsequent experiments. Forward and reverse primers specific to ROM were generated and used to amplify the target from first-strand cDNA in real-time PCR analysis. The cycle threshold method was used to deduce and confirm a reduction in the expression of this gene in KINE compared to CON cells (Figure 6).

### ROM expression is reduced in lung cancer cell lines

**Figure 7:**
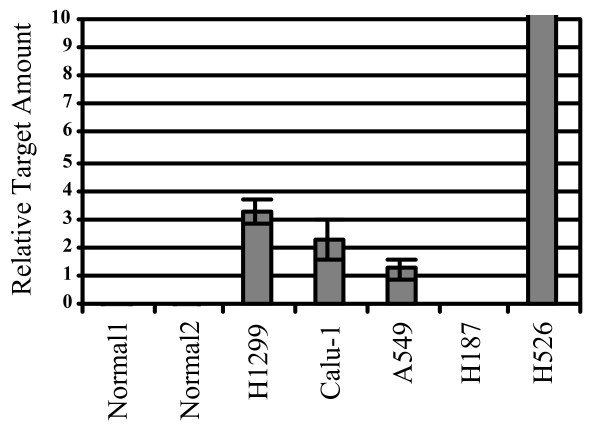
**The expression of ROM is downregulated in lungcancer cell lines**. Real-time PCR detected high levels of ROM in normal lung tissue and in the small cell lung cancer H526. The presence of ROM was, however, strongly reduced in several other lung cancer cell lines; H1299, Calu-1, A549 and H187.

Since the expression of ROM was downregulated in KINE which were significantly more invasive than CON cells, we investigated whether the expression of ROM may be modified in lung cancer cell lines. To test this, three non-small cell lung cancer cell lines, H1299, Calu-1, A549, two small cell lung cancer cell lines, H526 and H187, as well as two samples from normal lung tissue were analyzed by real-time PCR for the expression of ROM. All cancer cell lines apart from H526 were found to have reduced expression of ROM compared to the normal tissue samples indicating that ROM may play a role in the process of cell transformation (Figure 7).

## Discussion

The heterogeneity of the H460 large cell lung cancer cell line was investigated by selecting for chemokinetic cells from a CON population that demonstrated both chemokinesis and chemotaxis. Using Boyden chambers, cells that migrated under chemokinetic conditions were collected and their numbers expanded. Time-lapsed microscopy under isotropic conditions showed that KINE cells moved faster and changed directions more frequently than CON confirming their chemokinetic character. KINE cells which lacked stable focal adhesion were also less adhesive to culture plates compared to CON cells which had focal adhesions at the leading edge shown by phospho-Paxillin-tyr118 antibody labeling. Weak substrate adhesion in KINE cells may account for motile characteristics of rapid and random movement [16-19]. Furthermore, the selection for increased chemokinesis did not compromise the ability of KINE cells to chemotax. KINE cells were also significantly more invasive compared to CON.

These results underscore the importance of chemokinesis in cancer cell invasion suggesting that both chemokinetic and chemotactic abilities of cells should be evaluated when assessing invasive potential. Usually, chemotaxis alone is used to correlate with invasion data from *in vivo *and *in vitro *experiments. In checkerboard analyses, chemokinesis may constitute as high as 35% of total cell motility (both chemotaxis and chemokinesis) but is frequently eliminated in the subsequent evaluation of the molecular regulation of invasion [20]. This may introduce bias in the assessment of the relative importance of the different types of cell motility and cause attribution of molecular signals to the wrong motility type. It is possible that the chemokinetic cells are also chemotactic and represent the main cell cohort that migrated in the assays used.

Understanding the molecular process of chemotaxis is a work-in-progress in cancer, and there is even less understood about the molecular pathways and underlying processes in chemokinesis. Cancer cell chemotaxis downstream of the epidermal growth factor receptor (EGFR) pathway, involves the activation of cofilin by phospholipase Cγ (PLCγ). Cofilin, in turn, generates barbed ends on branched actin filaments and is sufficient to determine directionality of cell protrusion [21,22]. Activation of PI3K by EGFR is also relevant in motility inducing the phosphorylation of phosphtidylinositol-4, 5-biphosphate (PIP2) and catalyzing the formation of phosphtidylinositol-3, 4, 5-triphosphate (PIP3) at the plasma membrane. These inositol lipids are targets for pleckstrin-homology (PH) domain-containing proteins including nucleotide-exchange factors [23]. The exchange factors for Rho-like GTPases induce the recruitment and activation of Rac and Cdc24, which remodel the actin cytoskeleton [24-26] through effectors such as members of the neural Wiskott-Aldrich protein (N-WASP) family. These proteins activate Arp2/3-mediated dendritic nucleation and actin bundling to drive the formation of lamellae and filopodia [27]. Subsequent adhesion of these protrusions to the extracellular matrix generates both tensile and contractile forces; net forces favour the retraction of the trailing edge resulting in the forward movement of the cell [28,29].

Chemokinesis may involve a degree of stochasticity analogous to the symmetry breaking behaviour of *Saccharomyces cereviesiae *[30]. Yeast cells lacking the positional landmark Ras-family GTPase, Rsr1p, which interacts with Cdc42, are able to polarize, albeit randomly, towards a cortical site and undergo normal proliferation. The latter process, termed symmetry breaking is reliant upon the scaffold protein Bem1p, which recruits Cdc42 and promotes the local generation of more GTP-Cdc42 through a positive feedback mechanism. This process is thought to be promoted by stochastic fluctuations in the local concentrations of molecules. A feedback loop is initiated when local levels at a random cortical site exceed a threshold value. Similar stochastic processes may govern chemokinesis of cancer cells resulting in random turning behaviour.

This study shows that cancer cells capable of both chemotaxis and chemokinesis represent the highly motile cohort (faster speeds) that correlate well with invasiveness. We speculate chemokinesis in these cells may operate through an efficient stochastic process under isotropic conditions but in the presence of a strong gradient, their motility is convertible into chemotaxis. Therefore, the development of chemokinesis in highly motile cells complements the molecular machinery required for chemotaxis and does not necessarily inhibit the latter process. It follows that there may exist unique molecular determinants of chemokinesis that are separate from those that regulate chemotaxis. These determinants may also dictate hyperinvasiveness associated with the development of chemokinesis.

To investigate this further, we conducted gene chip microarray studies, which together with and real-time PCR analyses showed that a gene, reduced on-random Motile (ROM), was downregulated in chemokinetic cells. ROM encodes an adaptor protein [31] with several functional domains including a PDZ domain, a leucine-rich (LEU) and a PDZ domain binding region at the C-terminus. It has a short history in the literature; it was isolated on two occassions as a gene that was upregulated in the TH1 subset of cells and was called cytohesin-binding protein (CBP) [32] and cytohesin-binder and regulator (CYBR). The LEU region of CBP/CYBR/ROM was shown to bind cytohesin-1 and enhance the activity of ADP ribosylation factor-1 (ARF1) GTPase [33].

ROM has a closely related homologue known as GRP-1-associated-scaffold-protein (GRASP) sharing an overall 50% sequence homology and the presence of similar domains. GRASP has been shown to bind exchange factors for ARF GTPases, cytohesin-2 and GRP1 [34]. Tamalin, the mouse homologue of GRASP, was demonstrated to target glutamate receptors to the synapses and to bind proteins involved in establishing polarity in cells [35]. The functions of GRASP and Tamalin in plasma membrane targeting and polarization are highly suggestive that ROM may have similar roles. The expression of ROM was investigated in lung cancer cells and it was found that several cell lines were downregulated in the transcription of ROM compared to normal bronchial epithelial cells. A small cell lung cancer cell line, H187, did not have detectable levels of ROM transcripts.

## Conclusion

In summary, we tested the hypothesis that a cancer cell line may consist of subpopulations of cells that differed not only morphologically but also in their motility, invasiveness and molecular characteristics. Boyden chambers were used to isolate a subpopulation named KINE cells that were efficient at chemokinesis. However, we discovered that these cells were also chemotactic similar to the unselected CON population. The ability of the highly chemokinetic cells to chemotax is not surprising given that chemotaxis is not an efficient process in cancer cells, requiring steep gradients of chemoattractant [36]. KINE cells were less adhesive, more rounded in shape (less polarized), moved faster and were more invasive than CON cells. Microarray analysis and real-time PCR demonstrated that a gene, ROM, that was downregulated in KINE cells was also repressed in several lung cancer cell lines. This is suggestive of a role in cancer cell motility and invasion.

## Competing interests

The author(s) declare that they have no competing interests.

## Authors' contributions

LS, SF and FB participated in the design, funding, coordination, molecular genetic studies, statistical analyses and writing of the manuscript. FP, JC, JR participated in the design and experiments of the cell adhesion studies including localization of actin and paxillin. All authors have read and approved the final manuscript.

## Pre-publication history

The pre-publication history for this paper can be accessed here:



## Figures and Tables

**Table 1:** Checkerboard configuration for chemotaxis and chemokinesis. Details of assays are described in Materials and Method. Upper and lower Boyden chambers were filled with buffer containing various concentrations of serum as indicated. Chemokinetic combinations include A, E, F, I, J, K, M, N, O and P. Chemotactic conditions are B, C, D, G, H, and L.

**Lower Chamber**					
**UPPER**		**0% FBS**	**1.5% FBS**	**3% FBS**	**10% FBS**

	**0% FBS**	A	B	C	D
	**1.5% FBS**	E	F	G	H
	**3% FBS**	I	J	K	L
	**10% FBS**	M	N	O	P

**Table 2:** Checkerboard result for chemotaxis and chemokinesis. The number of cells that migrated across the filter was counted at the end of the 8 h assay. Plus (+) and minus (-) indicators show, respectively, the presence or absence of cells on the underside of the filter. The number of indicators (plus or minus) approximates the level of cell migration.

**Lower Chamber**					
**UPPER**		**0% FBS**	**1.5% FBS**	**3% FBS**	**10% FBS**

	**0 % FBS**	-	+++	+++++	+++++++
	**1.5% FBS**	+	+	+	+++
	**3% FBS**	+	+	++	+++
	**10% FBS**	+	+	++	++

**Table 3:** Parameter measurements of chemokinesis. Student's T-tests comparing parameters of motility between KINE and CON cells rejecting the Ho at P less than 0.05. Mean, Standard Deviation and P values are tabulated (* indicate significant difference; NS = non-significant difference).

H460 Cells	Total Path Length (μm)	Net Path Length (μm)	Directionality	Speed (μm/min)	Area (sq. μm)
CON *SE*	24.56 *+/-2.83*	11.87 *+/-3.75*	0.48 *+/-0.11*	0.33 *+/-0.02*	661.42 *+/-159.37*
KINE *SE*	33.11 *+/-4.71*	7.81 *+/-4.09*	0.23 *+/-0.11*	0.43 *+/-0.06*	413.60 *+/-83.37*
T-Test	*P = 0.014	P = 0.181 NS	*P = 0.018	*P = 0.014	*P = 0.025
