# Correction: Rapid chemokinetic movement and the invasive potential of lung cancer cells; a functional molecular study

**DOI:** 10.1186/1471-2407-7-32

**Published:** 2007-02-22

**Authors:** Kam-Meng Tchou-Wong, Sandra YY Fok, Jeffrey S Rubin, Fiona Pixley, John Condeelis, Filip Braet, William Rom, Lilian L Soon

**Affiliations:** 1 Departments of Medicine and Environmental Medicine, Division of Pulmonary and Critical Care Medicine, NYU School of Medicine, New York, NY 10016, USA; 2 Electron Microscope Unit, Australian Key Centre for Microscopy and Microanalysis, University of Sydney, Sydney, NSW, 2006, Australia; 3 Laboratory of Cellular and Molecular Biology, NCI, National Institutes of Health, Bethesda, MD 20892, USA; 4 Department of Anatomy and Structural Biology, Albert Einstein College of Medicine of Yeshiva University, Bronx, NY 10461, USA

## Abstract

This is a correction of an earlier published article.

Although Dr. Tchou-Wong and Dr. Rom were originally meant to be first and seventh authors, respectively, they were left off the authorship list when this manuscript was submitted to *BMC Cancer *[1]. We sincerely apologise to Dr. Tchou-Wong and Dr. Rom, and have reinstated them to their appropriate places within the author list.

Funding sources for this research include MO1-RR00096, RO1-ES0916 and NHMRC402510.

In addition, improper software conversion resulted in missing histogram bars in figure 7 of the article. This is corrected in figure 1.

## Pre-publication history

The pre-publication history for this paper can be accessed here:



## Figures and Tables

**Figure 1:**
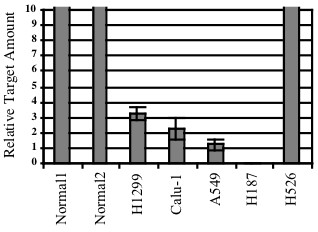
**The expression of ROM is downregulated in lung cancer cell lines**. Real-time PCR detected high levels of ROM in normal lung tissue and in the small cell lung cancer H526. The presence of ROM was, however, strongly reduced in several other lung cancer cell lines; H1299, Calu-1, A549 and H187.
